# Limitations of pupil diameter as a proxy for modes of locus coeruleus activity

**DOI:** 10.64898/2026.07.14.738598

**Published:** 2026-10-16

**Authors:** Lowell W. Thompson, Siddhartha Joshi, Joshua I. Gold

## Abstract

The locus coeruleus-norepinephrine (LC-NE) system plays multiple roles in higher brain function that are thought to depend on its mode of activation, which reflects relationships between baseline and evoked activation levels. These relationships are evident in both single-unit LC activity and proposed physiological proxies of LC-NE activity, such as pupil size. Here we used measurements in awake monkeys to show that the baseline-evoked relationships evident within these two different measures are unreliably coupled between them: baseline-evoked relationships of the pupil are not predictive of those in the LC, and vice versa. These results imply that pupil modulations, which can reflect LC-NE activity, should be used with caution to make inferences about “phasic” (moderate baseline, high evoked) and “tonic” (high baseline, low evoked) LC-NE activity modes that are thought to support different forms of information processing in the brain.

